# Crystal structure of *N*-{4-[(6-chloro­pyridin-3-yl)meth­oxy]phen­yl}-2,6-di­fluoro­benzamide

**DOI:** 10.1107/S2056989015023701

**Published:** 2016-01-01

**Authors:** Ying Liang, Li-Qiao Shi, Zi-Wen Yang

**Affiliations:** a Hubei Biopesticide Engineering Research Center, Hubei Academy of Agricultural Science, Wuhan 430064, People’s Republic of China

**Keywords:** crystal structure, amide derivative, pyridine, hydrogen bonding, π–π contacts

## Abstract

In the title compound, C_19_H_13_ClF_2_N_2_O_2_, the conformation of the N—H bond in the amide segment is *anti* to the C=O bond. The mol­ecule is not planar, with dihedral angles between the central benzene ring and the outer benzene and pyridyl rings of 73.35 (7) and 81.26 (6)°, respectively. A weak intra­molecular C—H⋯O hydrogen bond occurs. In the crystal, N—H⋯N, C—H⋯O and C—H⋯F hydrogen bonds lead to the formation of dimers. The N—H⋯N inversion dimers are linked by π–π contacts between adjacent pyridine rings [centroid–centroid = 3.8541 (12) Å] and C—H⋯π inter­actions. These contacts combine to stack the mol­ecules along the *a* axis.

## Abstract 2

The mol­ecular and crystal structure of *N*-{4-[(6-chloro­pyridin-3-yl)meth­oxy]phen­yl}-2,6-di­fluoro­benzamide is reported. The crystal packing is stabilized by N—H⋯N, C—H⋯O, C—H⋯F and C—H⋯π hydrogen bonds supplemented by offset π–π stacking inter­actions.

## Chemical context   

**Figure 1:**
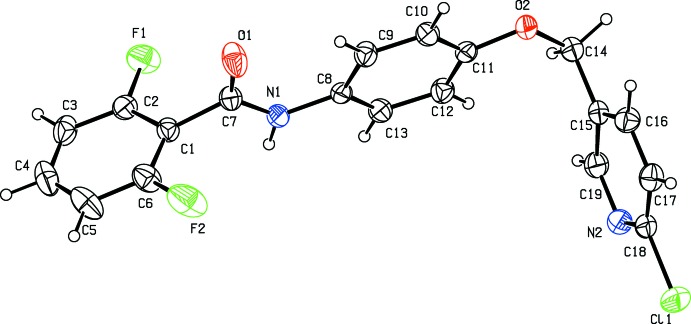
The structure of (I), showing 50% probability displacement ellipsoids and the atom-numbering scheme.

Amide derivatives show diverse biological properties, acting as insecticides (Liu *et al.*, 2004*a* ▸), fungicides (Liu *et al.*, 2004*b* ▸) and acaricides (Shiga *et al.*, 2003 ▸). Amides in regular commercial use include benzamide (flutolanil, fluopicolide), nicotinamide (boscalid) and thia­zole carboxamide (thifluzamide, ethaboxam). As a part of our work on the synthesis of novel fluorine-containing compounds with good biological activities, we report herein on the crystal structure of the title compound,(I), Fig. 1 ▸.
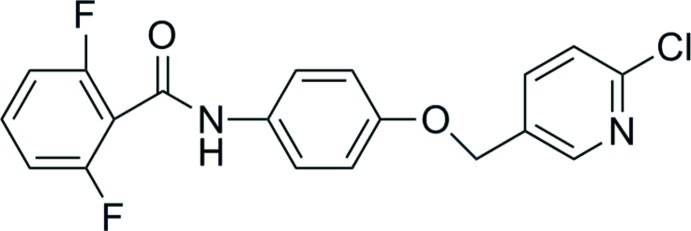


## Structural commentary   

The conformation of the N—H and the C=O bonds in the amide segment are *anti* to one another, similar to the conformation observed in another amide compound (Gowda *et al.*, 2010 ▸). The dihedral angle between the two benzene rings is 73.35 (6)° while that between the central benzene ring and the chloro-substituted pyridine ring is 81.26 (6). The amide residue C1/N1/C7/O1 lies close to the plane of the central benzene ring, making a dihedral angle of 8.73 (6)°. A weak intra­molecular C9—H9⋯O1 hydrogen bond (Table 1 ▸) contrib­utes to the planarity of this part of the mol­ecule.

## Supra­molecular features   

**Figure 2:**
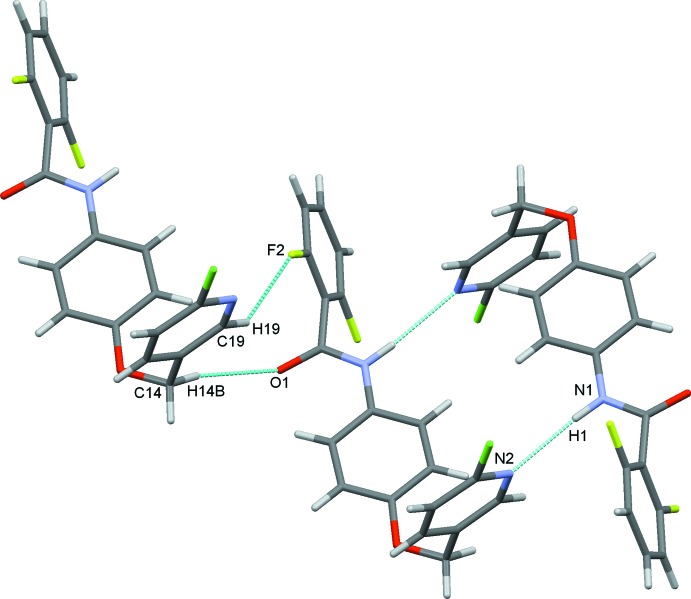
A pair of dimers with hydrogen bonds drawn as blue dashed lines.

**Figure 3:**
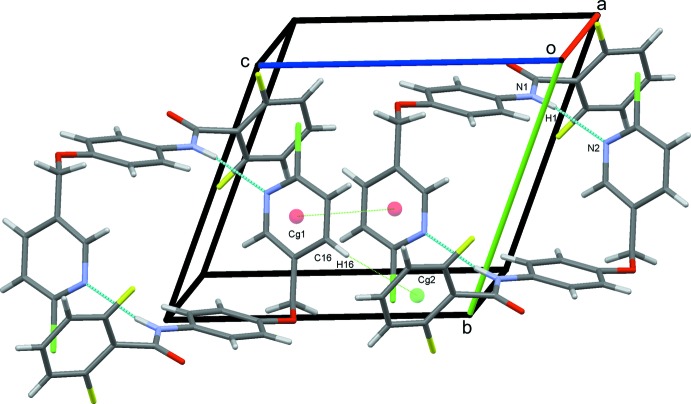
Chains of inversion dimers along the *c-*axis direction. Hydrogen bonds are drawn as dashed lines with π–π and C—H⋯π contacts shown as green dotted lines.

**Figure 4:**
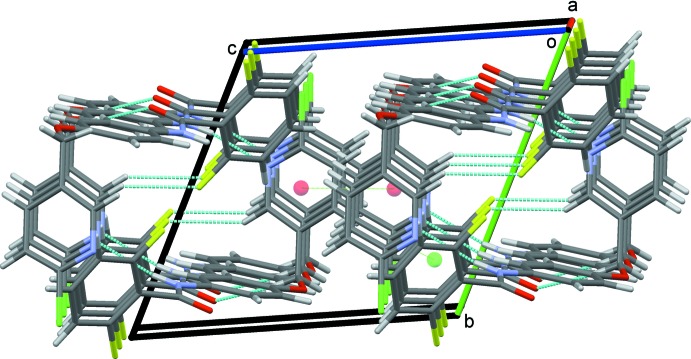
The overall packing for (I) viewed along the *a*-axis direction.

In the crystal structure, pairs of classical N1—H1⋯N2^i^ hydrogen bonds, Table 1 ▸, link the mol­ecules into inversion dimers and generate 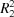
(22) rings (Bernstein *et al.*, 1995 ▸). C14—H14*B*⋯O1^ii^ and C19—H19⋯F2^ii^ hydrogen bonds also form dimers, which enclose an 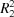
(10) ring motif, Fig. 2 ▸. The N—H⋯N dimers are linked into chains along the *c-*axis direction by π–π stacking inter­actions between adjacent pyridyl rings [*Cg*1⋯*Cg*1^iv^ = 3.8541 (12) Å; symmetry code: (iv) 1 − *x*, 1 − *y*, 1 − *z*] augmented by a weak C16—H16⋯*Cg*2 contact (*Cg*2 is the centroid of the C1–C6 benzene ring), Table 1 ▸, Fig. 3 ▸. These contacts combine to stack the mol­ecules along the *a* axis, Fig. 4 ▸.

## Synthesis and crystallization   

Tri­ethyl­amine (6mmol) was added dropwise to a stirred solution of 4-(6-chloro­pyridin-3-yl) meth­oxy aniline (5mmol) and 2,6-di­fluoro­benzoyl chloride (5mmol) in dry di­chloro­methane (20ml) at 275-277 K. The mixture was stirred at 283–288 K for 2 h, then washed with 0.5% hydro­chloric acid solution, and a saturated aqueous solution of sodium hydrogen carbonate, dried and evaporated. The residue was recrystallized from di­chloro­methane, giving colourless blocks of the title compound after three weeks.

## Database survey   

A search of the Cambridge Structural Database (Version 5.36 with three updates) (Groom & Allen, 2014 ▸) for *N*-(4-(pyridin-3-ylmeth­oxy)phen­yl)benzamide or its substituted derivatives gave no hits. However, structures of eight substituted 2,6-di­fluoro-*N*-phenyl­benzamide derivatives were found, see for example Cockroft *et al.* (2007 ▸); Spitaleri *et al.* (2004 ▸); Fun *et al.* (2010 ▸). Two structures of purely organic 3-(phen­oxy­meth­yl)pyridine derivatives have also been reported (Lakshminarayana *et al.*, 2009 ▸; Liu *et al.*, 2010 ▸) together with that of a cadmium complex of 4-[(6-chloro­pyridin-3-yl)meth­oxy]benz­oate, Li *et al.* (2007 ▸).

## Refinement   

Crystal data, data collection and structure refinement details are summarized in Table 2 ▸. The NH H atom was located in a difference Fourier map and freely refined. The C-bound H atoms were positioned geometrically and refined using a riding model with *d*(C—H) = 0.93–0.97 Å and *U*_iso_(H) = 1.2*U*_eq_(C).

## Supplementary Material

Crystal structure: contains datablock(s) global, I. DOI: 10.1107/S2056989015023701/sj5486sup1.cif

Structure factors: contains datablock(s) I. DOI: 10.1107/S2056989015023701/sj5486Isup2.hkl

Supporting information file. DOI: 10.1107/S2056989015023701/sj5486Isup3.cml

CCDC reference: 1441555

Additional supporting information:  crystallographic information; 3D view; checkCIF report

## Figures and Tables

**Table 1:** Hydrogen-bond geometry (Å, °) *Cg*2 is the centroid of the C1–C6 benzene ring.

*D*—H⋯*A*	*D*—H	H⋯*A*	*D*⋯*A*	*D*—H⋯*A*
C9—H9⋯O1	0.93	2.27	2.863 (2)	121
N1—H1⋯N2^i^	0.88 (2)	2.24 (2)	3.109 (2)	170.6 (18)
C19—H19⋯F2^ii^	0.93	2.54	3.309 (2)	140
C14—H14*B*⋯O1^ii^	0.97	2.42	3.344 (3)	160
C16—H16⋯*Cg*2^iii^	0.93	2.99	3.912 (2)	173

Symmetry codes: (i) 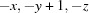; (ii) 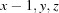; (iii) 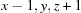.

**Table 2:** Experimental details

Crystal data	
Chemical formula	C_19_H_13_ClF_2_N_2_O_2_
*M*_r_	374.76
Crystal system, space group	Triclinic, *P* 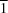
Temperature (K)	298
*a*, *b*, *c* (Å)	8.8173 (11), 10.7036 (13), 10.8452 (14)
α, β, γ (°)	61.939 (2), 77.597 (2), 69.636 (2)
*V* (Å^3^)	845.15 (18)
*Z*	2
Radiation type	Mo *K*α
μ (mm^−1^)	0.26
Crystal size (mm)	0.16 × 0.12 × 0.10

Data collection	
Diffractometer	Bruker SMART APEX CCD area detector
Absorption correction	Multi-scan (*SADABS*; Sheldrick, 2001 ▸)
*T*_min_, *T*_max_	0.959, 0.974
No. of measured, independent and observed [*I* > 2σ(*I*)] reflections	5474, 3271, 2886
*R*_int_	0.029
(sin θ/λ)_max_ (Å^−1^)	0.617

Refinement	
*R*[*F* ^2^ > 2σ(*F* ^2^)], *wR*(*F* ^2^), *S*	0.045, 0.116, 1.06
No. of reflections	3271
No. of parameters	239
H-atom treatment	H atoms treated by a mixture of independent and constrained refinement
Δρ_max_, Δρ_min_ (e Å^−3^)	0.21, −0.23

Computer programs: *SMART* and *SAINT* (Bruker, 2000 ▸), *SHELXS97*, *SHELXL97* and *SHELXTL* (Sheldrick, 2008 ▸) and *Mercury* (Macrae *et al.*, 2008 ▸).

## supplementary crystallographic information

### Crystal data

**Table:**

C_19_H_13_ClF_2_N_2_O_2_	*Z* = 2
*M**_r_* = 374.76	*F*(000) = 384
Triclinic, *P*1	*D*_x_ = 1.473 Mg m^−^^3^
Hall symbol: -P 1	Mo *K*α radiation, λ = 0.71073 Å
*a* = 8.8173 (11) Å	Cell parameters from 2810 reflections
*b* = 10.7036 (13) Å	θ = 2.3–28.3°
*c* = 10.8452 (14) Å	µ = 0.26 mm^−^^1^
α = 61.939 (2)°	*T* = 298 K
β = 77.597 (2)°	Block, colorless
γ = 69.636 (2)°	0.16 × 0.12 × 0.10 mm
*V* = 845.15 (18) Å^3^	

### Data collection

**Table:**

Bruker SMART APEX CCD area-detector diffractometer	3271 independent reflections
Radiation source: fine-focus sealed tube	2886 reflections with *I* > 2σ(*I*)
Graphite monochromator	*R*_int_ = 0.029
φ and ω scans	θ_max_ = 26.0°, θ_min_ = 2.1°
Absorption correction: multi-scan (*SADABS*; Sheldrick, 2001)	*h* = −10→10
*T*_min_ = 0.959, *T*_max_ = 0.974	*k* = −13→13
5474 measured reflections	*l* = −12→13

### Refinement

**Table:**

Refinement on *F*^2^	Primary atom site location: structure-invariant direct methods
Least-squares matrix: full	Secondary atom site location: difference Fourier map
*R*[*F*^2^ > 2σ(*F*^2^)] = 0.045	Hydrogen site location: inferred from neighbouring sites
*wR*(*F*^2^) = 0.116	H atoms treated by a mixture of independent and constrained refinement
*S* = 1.06	*w* = 1/[σ^2^(*F*_o_^2^) + (0.0526*P*)^2^ + 0.196*P*] where *P* = (*F*_o_^2^ + 2*F*_c_^2^)/3
3271 reflections	(Δ/σ)_max_ < 0.001
239 parameters	Δρ_max_ = 0.21 e Å^−^^3^
0 restraints	Δρ_min_ = −0.23 e Å^−^^3^

### Special details

**Table:**

Geometry. All e.s.d.'s (except the e.s.d. in the dihedral angle between two l.s. planes) are estimated using the full covariance matrix. The cell e.s.d.'s are taken into account individually in the estimation of e.s.d.'s in distances, angles and torsion angles; correlations between e.s.d.'s in cell parameters are only used when they are defined by crystal symmetry. An approximate (isotropic) treatment of cell e.s.d.'s is used for estimating e.s.d.'s involving l.s. planes.
Refinement. Refinement of *F*^2^ against ALL reflections. The weighted *R*-factor *wR* and goodness of fit *S* are based on *F*^2^, conventional *R*-factors *R* are based on *F*, with *F* set to zero for negative *F*^2^. The threshold expression of *F*^2^ > σ(*F*^2^) is used only for calculating *R*-factors(gt) *etc*. and is not relevant to the choice of reflections for refinement. *R*-factors based on *F*^2^ are statistically about twice as large as those based on *F*, and *R*- factors based on ALL data will be even larger.

### Fractional atomic coordinates and isotropic or equivalent isotropic displacement parameters (Å^2^)

**Table:**

	*x*	*y*	*z*	*U*_iso_*/*U*_eq_	
C1	0.7630 (2)	0.2167 (2)	−0.02643 (18)	0.0427 (4)	
C2	0.8270 (2)	0.1167 (2)	−0.08394 (19)	0.0478 (4)	
C3	0.9422 (3)	0.1317 (3)	−0.1918 (2)	0.0611 (6)	
H3	0.9816	0.0618	−0.2276	0.073*	
C4	0.9982 (3)	0.2526 (3)	−0.2461 (2)	0.0706 (7)	
H4	1.0773	0.2644	−0.3195	0.085*	
C5	0.9403 (3)	0.3566 (3)	−0.1949 (2)	0.0750 (7)	
H5	0.9787	0.4388	−0.2327	0.090*	
C6	0.8239 (3)	0.3360 (2)	−0.0861 (2)	0.0602 (5)	
C7	0.6521 (2)	0.1886 (2)	0.10413 (18)	0.0432 (4)	
C8	0.3644 (2)	0.21024 (17)	0.18987 (17)	0.0391 (4)	
C9	0.3813 (2)	0.1688 (2)	0.32952 (18)	0.0462 (4)	
H9	0.4817	0.1503	0.3590	0.055*	
C10	0.2489 (2)	0.1551 (2)	0.42425 (18)	0.0461 (4)	
H10	0.2609	0.1273	0.5176	0.055*	
C11	0.0990 (2)	0.18196 (17)	0.38294 (18)	0.0408 (4)	
C12	0.0809 (2)	0.2233 (2)	0.24416 (19)	0.0470 (4)	
H12	−0.0198	0.2422	0.2149	0.056*	
C13	0.2140 (2)	0.2363 (2)	0.14935 (18)	0.0461 (4)	
H13	0.2019	0.2631	0.0563	0.055*	
C14	−0.1851 (2)	0.2030 (2)	0.4525 (2)	0.0497 (5)	
H14A	−0.2488	0.1521	0.5341	0.060*	
H14B	−0.1861	0.1737	0.3806	0.060*	
C15	−0.2607 (2)	0.36755 (19)	0.40005 (18)	0.0414 (4)	
C16	−0.2680 (2)	0.4370 (2)	0.4829 (2)	0.0498 (5)	
H16	−0.2264	0.3819	0.5710	0.060*	
C17	−0.3362 (2)	0.5864 (2)	0.4347 (2)	0.0506 (5)	
H17	−0.3418	0.6348	0.4885	0.061*	
C18	−0.3961 (2)	0.6622 (2)	0.3043 (2)	0.0479 (4)	
C19	−0.3260 (2)	0.4551 (2)	0.2721 (2)	0.0503 (5)	
H19	−0.3230	0.4098	0.2161	0.060*	
Cl1	−0.48341 (8)	0.85162 (6)	0.23999 (7)	0.0783 (2)	
F1	0.77345 (18)	−0.00346 (13)	−0.02627 (13)	0.0741 (4)	
F2	0.7661 (2)	0.43493 (16)	−0.03160 (16)	0.1031 (6)	
N1	0.49454 (18)	0.22686 (16)	0.08547 (16)	0.0430 (4)	
H1	0.468 (2)	0.265 (2)	−0.001 (2)	0.052*	
N2	−0.3942 (2)	0.60203 (18)	0.22226 (16)	0.0537 (4)	
O1	0.71012 (17)	0.1345 (2)	0.21658 (15)	0.0727 (5)	
O2	−0.02172 (15)	0.16015 (14)	0.48849 (13)	0.0495 (3)	

### Atomic displacement parameters (Å^2^)

**Table:**

	*U*^11^	*U*^22^	*U*^33^	*U*^12^	*U*^13^	*U*^23^
C1	0.0410 (9)	0.0482 (10)	0.0375 (9)	−0.0112 (8)	−0.0013 (7)	−0.0189 (8)
C2	0.0514 (11)	0.0483 (10)	0.0415 (10)	−0.0084 (8)	−0.0054 (8)	−0.0207 (8)
C3	0.0524 (12)	0.0763 (15)	0.0463 (11)	0.0010 (11)	−0.0033 (9)	−0.0335 (11)
C4	0.0484 (12)	0.116 (2)	0.0456 (12)	−0.0288 (13)	0.0066 (9)	−0.0342 (13)
C5	0.0860 (17)	0.1005 (19)	0.0517 (13)	−0.0615 (15)	0.0073 (12)	−0.0240 (13)
C6	0.0785 (15)	0.0630 (13)	0.0489 (11)	−0.0310 (11)	0.0043 (10)	−0.0278 (10)
C7	0.0470 (10)	0.0465 (9)	0.0385 (9)	−0.0125 (8)	−0.0001 (8)	−0.0220 (8)
C8	0.0434 (9)	0.0330 (8)	0.0364 (9)	−0.0069 (7)	0.0014 (7)	−0.0159 (7)
C9	0.0425 (10)	0.0540 (10)	0.0408 (10)	−0.0085 (8)	−0.0032 (8)	−0.0230 (8)
C10	0.0508 (11)	0.0483 (10)	0.0347 (9)	−0.0076 (8)	−0.0026 (8)	−0.0188 (8)
C11	0.0453 (10)	0.0322 (8)	0.0391 (9)	−0.0062 (7)	0.0016 (7)	−0.0160 (7)
C12	0.0423 (10)	0.0523 (10)	0.0444 (10)	−0.0092 (8)	−0.0041 (8)	−0.0219 (9)
C13	0.0512 (11)	0.0480 (10)	0.0355 (9)	−0.0090 (8)	−0.0030 (8)	−0.0188 (8)
C14	0.0457 (10)	0.0462 (10)	0.0515 (11)	−0.0141 (8)	0.0055 (8)	−0.0194 (9)
C15	0.0353 (9)	0.0447 (9)	0.0424 (9)	−0.0120 (7)	0.0045 (7)	−0.0198 (8)
C16	0.0479 (11)	0.0540 (11)	0.0457 (10)	−0.0101 (8)	−0.0079 (8)	−0.0214 (9)
C17	0.0483 (11)	0.0552 (11)	0.0573 (12)	−0.0160 (9)	0.0002 (9)	−0.0321 (10)
C18	0.0379 (9)	0.0427 (9)	0.0550 (11)	−0.0123 (7)	0.0062 (8)	−0.0179 (9)
C19	0.0544 (11)	0.0524 (11)	0.0444 (10)	−0.0118 (9)	0.0011 (8)	−0.0254 (9)
Cl1	0.0732 (4)	0.0415 (3)	0.0948 (5)	−0.0080 (2)	0.0032 (3)	−0.0186 (3)
F1	0.1111 (11)	0.0569 (7)	0.0632 (8)	−0.0301 (7)	0.0031 (7)	−0.0322 (6)
F2	0.1807 (18)	0.0716 (9)	0.0786 (10)	−0.0650 (11)	0.0283 (10)	−0.0444 (8)
N1	0.0453 (9)	0.0451 (8)	0.0327 (8)	−0.0082 (6)	−0.0008 (6)	−0.0161 (7)
N2	0.0541 (10)	0.0527 (9)	0.0428 (9)	−0.0079 (7)	−0.0019 (7)	−0.0173 (7)
O1	0.0500 (8)	0.1190 (14)	0.0444 (8)	−0.0210 (9)	−0.0008 (7)	−0.0348 (9)
O2	0.0436 (7)	0.0507 (7)	0.0411 (7)	−0.0063 (6)	0.0034 (5)	−0.0170 (6)

### Geometric parameters (Å, º)

**Table:**

C1—C6	1.374 (3)	C11—O2	1.377 (2)
C1—C2	1.382 (2)	C11—C12	1.383 (2)
C1—C7	1.504 (2)	C12—C13	1.384 (2)
C2—F1	1.344 (2)	C12—H12	0.9300
C2—C3	1.361 (3)	C13—H13	0.9300
C3—C4	1.366 (3)	C14—O2	1.432 (2)
C3—H3	0.9300	C14—C15	1.508 (2)
C4—C5	1.368 (4)	C14—H14A	0.9700
C4—H4	0.9300	C14—H14B	0.9700
C5—C6	1.374 (3)	C15—C19	1.371 (3)
C5—H5	0.9300	C15—C16	1.391 (2)
C6—F2	1.347 (2)	C16—C17	1.368 (3)
C7—O1	1.217 (2)	C16—H16	0.9300
C7—N1	1.336 (2)	C17—C18	1.372 (3)
C8—C13	1.381 (3)	C17—H17	0.9300
C8—C9	1.390 (2)	C18—N2	1.316 (2)
C8—N1	1.423 (2)	C18—Cl1	1.7328 (19)
C9—C10	1.379 (2)	C19—N2	1.345 (2)
C9—H9	0.9300	C19—H19	0.9300
C10—C11	1.378 (3)	N1—H1	0.88 (2)
C10—H10	0.9300		
			
C6—C1—C2	115.21 (17)	C11—C12—C13	119.36 (17)
C6—C1—C7	121.35 (16)	C11—C12—H12	120.3
C2—C1—C7	122.93 (16)	C13—C12—H12	120.3
F1—C2—C3	119.06 (18)	C8—C13—C12	121.47 (16)
F1—C2—C1	117.06 (16)	C8—C13—H13	119.3
C3—C2—C1	123.86 (19)	C12—C13—H13	119.3
C2—C3—C4	118.0 (2)	O2—C14—C15	111.33 (15)
C2—C3—H3	121.0	O2—C14—H14A	109.4
C4—C3—H3	121.0	C15—C14—H14A	109.4
C3—C4—C5	121.5 (2)	O2—C14—H14B	109.4
C3—C4—H4	119.3	C15—C14—H14B	109.4
C5—C4—H4	119.3	H14A—C14—H14B	108.0
C4—C5—C6	118.1 (2)	C19—C15—C16	116.97 (17)
C4—C5—H5	121.0	C19—C15—C14	122.83 (17)
C6—C5—H5	121.0	C16—C15—C14	120.19 (16)
F2—C6—C1	116.98 (18)	C17—C16—C15	119.99 (17)
F2—C6—C5	119.7 (2)	C17—C16—H16	120.0
C1—C6—C5	123.4 (2)	C15—C16—H16	120.0
O1—C7—N1	125.21 (17)	C16—C17—C18	117.59 (17)
O1—C7—C1	118.93 (16)	C16—C17—H17	121.2
N1—C7—C1	115.86 (15)	C18—C17—H17	121.2
C13—C8—C9	118.73 (16)	N2—C18—C17	124.97 (17)
C13—C8—N1	117.80 (15)	N2—C18—Cl1	116.22 (15)
C9—C8—N1	123.47 (16)	C17—C18—Cl1	118.81 (15)
C10—C9—C8	119.84 (17)	N2—C19—C15	124.31 (17)
C10—C9—H9	120.1	N2—C19—H19	117.8
C8—C9—H9	120.1	C15—C19—H19	117.8
C11—C10—C9	121.11 (16)	C7—N1—C8	127.66 (15)
C11—C10—H10	119.4	C7—N1—H1	116.4 (13)
C9—C10—H10	119.4	C8—N1—H1	115.9 (13)
O2—C11—C10	115.23 (15)	C18—N2—C19	116.17 (17)
O2—C11—C12	125.25 (16)	C11—O2—C14	118.83 (14)
C10—C11—C12	119.49 (16)		
			
C6—C1—C2—F1	178.43 (17)	C10—C11—C12—C13	0.3 (3)
C7—C1—C2—F1	6.5 (3)	C9—C8—C13—C12	0.6 (3)
C6—C1—C2—C3	0.0 (3)	N1—C8—C13—C12	−179.67 (16)
C7—C1—C2—C3	−171.90 (18)	C11—C12—C13—C8	−0.6 (3)
F1—C2—C3—C4	−178.15 (19)	O2—C14—C15—C19	123.96 (19)
C1—C2—C3—C4	0.2 (3)	O2—C14—C15—C16	−56.7 (2)
C2—C3—C4—C5	−0.4 (3)	C19—C15—C16—C17	−0.8 (3)
C3—C4—C5—C6	0.3 (4)	C14—C15—C16—C17	179.80 (17)
C2—C1—C6—F2	−179.11 (18)	C15—C16—C17—C18	0.3 (3)
C7—C1—C6—F2	−7.1 (3)	C16—C17—C18—N2	0.6 (3)
C2—C1—C6—C5	−0.1 (3)	C16—C17—C18—Cl1	179.99 (14)
C7—C1—C6—C5	171.9 (2)	C16—C15—C19—N2	0.7 (3)
C4—C5—C6—F2	178.9 (2)	C14—C15—C19—N2	−179.97 (17)
C4—C5—C6—C1	0.0 (4)	O1—C7—N1—C8	−1.5 (3)
C6—C1—C7—O1	−77.8 (3)	C1—C7—N1—C8	178.76 (15)
C2—C1—C7—O1	93.7 (2)	C13—C8—N1—C7	−170.21 (16)
C6—C1—C7—N1	102.0 (2)	C9—C8—N1—C7	9.5 (3)
C2—C1—C7—N1	−86.6 (2)	C17—C18—N2—C19	−0.7 (3)
C13—C8—C9—C10	−0.3 (3)	Cl1—C18—N2—C19	179.84 (14)
N1—C8—C9—C10	−179.98 (16)	C15—C19—N2—C18	0.1 (3)
C8—C9—C10—C11	0.0 (3)	C10—C11—O2—C14	172.31 (14)
C9—C10—C11—O2	178.18 (16)	C12—C11—O2—C14	−9.6 (2)
C9—C10—C11—C12	0.0 (3)	C15—C14—O2—C11	−78.49 (19)
O2—C11—C12—C13	−177.66 (16)		

### Hydrogen-bond geometry (Å, º)

Cg2 is the centroid of the C1–C6 benzene ring.

**Table:**

*D*—H···*A*	*D*—H	H···*A*	*D*···*A*	*D*—H···*A*
C9—H9···O1	0.93	2.27	2.863 (2)	121
N1—H1···N2^i^	0.88 (2)	2.24 (2)	3.109 (2)	170.6 (18)
C19—H19···F2^ii^	0.93	2.54	3.309 (2)	140
C14—H14*B*···O1^ii^	0.97	2.42	3.344 (3)	160
C16—H16···*Cg*2^iii^	0.93	2.99	3.912 (2)	173

Symmetry codes: (i) −*x*, −*y*+1, −*z*; (ii) *x*−1, *y*, *z*; (iii) *x*−1, *y*, *z*+1.

## References

[bb1] Bernstein, J., Davis, R. E., Shimoni, L. & Chang, N.-L. (1995). *Angew. Chem. Int. Ed. Engl.* **34**, 1555–1573.

[bb2] Bruker (2000). *SMART*, *SAINT* and *SADABS*. Bruker AXS Inc., Madison, Wisconsin, USA.

[bb3] Cockroft, S. L., Perkins, J., Zonta, C., Adams, H., Spey, S. E., Low, C. M. R., Vinter, J. G., Lawson, K. R., Urch, C. J. & Hunter, C. A. (2007). *Org. Biomol. Chem.* **5**, 1062–1080. 10.1039/b617576g 17377660

[bb4] Fun, H.-K., Goh, J. H., Gowda, J., Khader, A. M. & Kalluraya, B. (2010). *Acta Cryst.* E**66**, o3192. 10.1107/S1600536810046507 PMC3011460 21589486

[bb5] Gowda, B. T., Tokarčík, M., Shakuntala, K., Kožíšek, J. & Fuess, H. (2010). *Acta Cryst.* E**66**, o1529–o1530. 10.1107/S1600536810019999 PMC3006934 21587779

[bb6] Groom, C. R. & Allen, F. H. (2014). *Angew. Chem. Int. Ed.* **53**, 662–671. 10.1002/anie.201306438 24382699

[bb7] Lakshminarayana, B. N., Prasad, J. S., Venu, T. D., Manuprasad, B. K., Sridhar, M. A. & Shashikanth, S. (2009). *Mol. Cryst. Liq. Cryst.* **515**, 207–214.

[bb8] Li, S.-L., Liu, J. & Liu, Y.-Y. (2007). *Acta Cryst.* E**63**, m2956.

[bb9] Liu, C. L., Li, L. & Li, Z. M. (2004*a*). *Bioorg. Med. Chem.* **12**, 2825–2830. 10.1016/j.bmc.2004.03.050 15142542

[bb10] Liu, C. L., Li, Z. M. & Zhong, B. (2004*b*). *J. Fluor. Chem.* **125**, 1287–1290.

[bb11] Liu, X.-H., Liu, H.-F., Shen, X., Song, B.-A., Bhadury, P. S., Zhu, H.-L., Liu, J.-X. & Qi, X.-B. (2010). *Bioorg. Med. Chem. Lett.* **20**, 4163–4167. 10.1016/j.bmcl.2010.05.080 20538457

[bb12] Macrae, C. F., Bruno, I. J., Chisholm, J. A., Edgington, P. R., McCabe, P., Pidcock, E., Rodriguez-Monge, L., Taylor, R., van de Streek, J. & Wood, P. A. (2008). *J. Appl. Cryst.* **41**, 466–470.

[bb13] Sheldrick, G. M. (2001). *SADABS*. University of Göttingen, Germany.

[bb14] Sheldrick, G. M. (2008). *Acta Cryst.* A**64**, 112–122. 10.1107/S0108767307043930 18156677

[bb15] Shiga, Y., Okada, I. & Fukuchi, T. (2003). *J. Pestic. Sci.* **28**, 310–312.

[bb16] Spitaleri, A., Hunter, C. A., McCabe, J. F., Packer, M. J. & Cockroft, S. L. (2004). *CrystEngComm*, **6**, 490–493.

